# External Quality Assessment Program for SARS‐COV‐2 Molecular Detection in Pakistan

**DOI:** 10.1111/irv.13316

**Published:** 2024-07-11

**Authors:** Nazish Badar, Aamer Ikram, Muhammad Salman, Sidra Saeed, Hamza Ahmed Mirza, Abdul Ahad, Asiya Ashraf, Umer Farooq

**Affiliations:** ^1^ Public Health Laboratories Division National Institute of Health Islamabad Pakistan; ^2^ Executive Director Office National Institute of Health Islamabad Pakistan; ^3^ Animal Sciences Institute National Agricultural Research Center Islamabad Pakistan

**Keywords:** external quality assurance, Pakistan, SARS CoV‐2

## Abstract

**Introduction:**

Amid coronavirus disease 2019 (COVID‐19) pandemic, accurate detection of severe acute respiratory syndrome coronavirus 2 (SARS‐CoV‐2) is critical for diagnosis management and breaking down transmission chains. We designed a national external quality assessment panel (EQAP) for SARS‐CoV‐2 molecular detection comprising working laboratories nationwide.

**Methods:**

A molecular diagnostic EQA panel that consists of five samples for SARS CoV‐2 testing was distributed to 141 public and private sector laboratories across country. These samples contain different concentrations of SARS‐CoV‐2 to evaluate the sensitivity of commercial kits available.

**Results:**

Sensitivity among public and private sector laboratories was variable, particularly lower SARS‐CoV‐2 concentrations significantly increased the risk of false‐negative tests, whereas Ct values of accurately tested SARS‐CoV‐2 specimens increased as concentration decreased. These findings highlighted that performance of used commercial kits was not significantly correlated to various extraction or PCR methods.

**Conclusion:**

This study highlights the need for a national external quality assessment panel (EQAP) in the country to improve the quality of the healthcare system while ensuring the accuracy and reliability of results. Furthermore, EQAPs can help laboratories meet accreditation and regulatory requirements. However, continued participation in EQAP is recommended for quality enhancement of laboratories.

## 1 Introduction

Severe acute respiratory syndrome coronavirus (SARS‐CoV‐2) had 7,010,568 million mortality out of 773,819,856 confirm cases (CFR 0.9%) globally as of December 31, 2023 [1]. Pakistan reported 30.7 k deaths out of 1.6 million confirm cases (CFR 1.9%), of SARS‐CoV‐2. Since SARS‐CoV‐2 was reported as the cause of coronavirus disease 2019, detection of the virus from clinical samples through commercial kits has been increased. Simultaneously, expanding SARS‐CoV‐2 diagnostic testing has become a primary public health goal for disease control and management. In clinical practice, robust detection of acute SARS‐CoV‐2 infected individuals by real‐time reverse transcription polymerase chain reaction (rtRT‐PCR) is crucial for clinical management, surveillance, and interrupting the chain of transmission [2]. As a result, laboratories must evaluate several diagnostic assays and integrate them into laboratory operations at the same time.

The importance of accurate diagnosis and quality assurance methods cannot be overstated. External quality assessments (EQAs) conducted by proficiency testing organizations, designed to assess laboratories' ability to identify a pathogenic agent at a clinically relevant level, are especially well suited to meet these standards defined by World Health Organization (WHO) [1, 3]. Furthermore, standardized EQAP samples allow a facility to analyze specific components of a test without relying entirely on the manufacturer's performance data. Government and public health laboratories, on the other hand, are constrained in their ability to execute the volume of tests required. As a result, patient identification and isolation, contact tracing, and even symptomatic patient treatment may be compromised. So, there has been a demand for rapid expansion of diagnostic testing for emerging diseases into private sector laboratories, allowing for increased test volume and shorter turnaround times (TAT). In Pakistan, the government has supported the use of nucleic acid amplification tests (NAATs), such as PCR, by enacting new policies such as in vitro diagnostic (IVD) emergency approval. However, NAATs are employed in various settings, including several new lab services, and their quality and diagnostic performance have not been well verified [4, 5, 6].

Furthermore, different laboratories use different nucleic acid extraction procedures, NAAT reagents, and testing platforms. EQA is critical to ensuring accurate test results, particularly when employing diagnostic kits for newly emerging diseases [4]. For this unique virus, the WHO has also encouraged laboratories to engage in EQA schemes [1].

EQA are well‐established strategy for enhancing and supporting diagnostic accuracy for clinical management and surveillance [7, 8]. Lately, several countries have conducted EQA for SARS‐CoV‐2 molecular testing and found potential flaws in nucleic acid extraction and PCR reagent kits [5, 9, 10, 11, 12]. Therefore, National Influenza Centre, virology department, National Institute of Health (NIH) Pakistan, designed a national molecular diagnostic EQA testing panel for SARS‐CoV‐2 testing. Participating laboratories were selected from public and private sectors during all EQA panels. During this study, the purpose was to assess the laboratory's diagnostic accuracy for SARS‐CoV‐2 detection countrywide.

## 2 Materials and Methods

### 2.1 COVID‐19 Participating Laboratories

During 2020–2022, the NIH registered public and private sectors SARS‐CoV‐2 testing laboratories for monitoring testing capacity across country. Participating laboratories were enrolled from that prepared list during four EQA panels. The public sector (provincial reference, influenza sentinel site, health care facilities) and private sector laboratories involved in molecular testing in Pakistan. First panel was sent on August 18, 2020, followed by second panel on December 17, 2020, third panel on April 7, 2021, and fourth panel on November 10, 2021.

### 2.2 EQA Panel Composition and Sample Preparation

**FIGURE 1:**
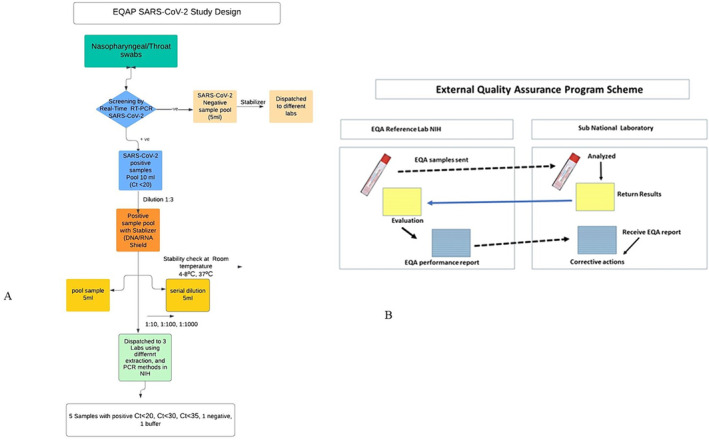
(A) Flowchart of EQAP SARS‐CoV‐2 study design. (B) Graphical presentation of working procedures.

The EQA samples were prepared (Figure 1A) under the prescribed standard operating procedures, detailed as follows:

Nasopharyngeal/throat swabs samples were collected from SARS‐CoV‐2 suspected individuals (Figure 1B). The screening was done by real‐time RT‐PCR at the reference lab. We selected positive (Ct value < 20) and negative samples by pooling method (“Pooling” refers to the practice of combining swab samples collected from multiple individuals before the RNA extraction step) [13, 14] for assessment of lab quality through EQAP. In the first step, negative and positive sample pool were prepared whereas positive samples having Ct value ≤ 20 were selected for panel preparation.

The negative and positive samples were stabilized (1:3) with DNA/RNA shield and underwent repeated stability tests by storing at different temperatures such as room temperature, 4°C–8°C and 37°C for 48 h. Furthermore, serial dilutions were prepared from a positive sample pool with stabilizer in 1:10, 1:100, and 1:1000 in Dulbecco's Modified Eagle's Medium (DMEM).

Before dispatch, these samples were tested in three different laboratories at the NIH using different extraction and PCR methods for screening SARS‐CoV‐2 commercial kits targeting E and N‐gene‐based assay, following WHO recommendations.

In the next step, the EQA panel for SARS‐CoV‐2 was prepared comprising five samples including three positives and two negatives. Positive samples having different Ct values for SARS‐CoV‐2 strong to borderline such as Ct < 20, Ct < 30, and Ct < 35 to check the sensitivity of different commercial kits for diagnostics (Table S1) where as one negative sample was from the pool of negative samples prepared with stabilizer and second was placebo consisting of DMEM buffer. This panel was dispatched to private and public sector laboratories at ambient temperature to assess the sensitivity of SARS‐CoV‐2 testing along with instructions of handling the samples and reporting results both in hard form and electronically (Figure S1).

According to the analysis of EQA panel criterion, selected laboratories with concordant result ≥ 80% will be considered as pass. These samples should be considered as unknown and tested as per their routine screening procedures, in line with manufacturer guidelines. To ensure uniform interpretation, a sample was scored as positive if at least one SARS‐CoV‐2 test gene resulted in a positive result. This 80% was selected in line with other studies such as Mögling et al. [7]. Participants in the EQAP were only asked to report whether or not their samples tested positive, negative, or undetermined/inconclusive for SARS‐CoV‐2. Laboratories were asked to provide information such as extraction platform and different rtRT‐PCR commercial kits used for SARS‐CoV‐2 screening (Table 1). After completing the EQAP testing, users could submit a result return form electronically.

**TABLE 1:** Comparison of PCR kits/target regions and EQAP samples.

Commercial assays company	Commercial kits	Genome target	No. of laboratories	Sample 1 (pool)	Sample 2 (1:10)	Sample 3 (1:100)	Sample 4 (buffer/negative)	Sample 5 (negative)
Mygo Pro 32	Anatolia	E, ORF1ab	7	100%	100%	100%	80%	100%
Sars‐CoV‐19	Sars‐CoV‐Florescent PCR Kit	ORF1, N	31	100%	100%	100%	80%	100%
Taq Path	Taqpath Covid 19‐PCR Kit	N, S, ORF	3	100%	100%	100%	60%	100%
Shingai Biotech	Life River	E, N, ORF 1ab	20	100%	100%	80%	60%	100%
Systaaq	Systaaq 2019‐Novel Coronavirus	N, RDRP	24	80%	100%	80%	60%	100%
Zeesan Sars Kit	Sars‐CoV‐2 Test Kit (Real‐Time PCR)‐Dry	ORF 1ab, N	14	100%	80%	100%	60%	100%
Argene	Argene	RDRP, N	2	100%	60%	60%	100%	80%
Meccura	Sars‐CoV‐Florescent PCR Kit	ORF1, E, N	19	60%	100%	100%	60%	80%
Visure	Systaaq 2019‐Novel Coronavirus	ORF1ab, N	21	100%	100%	80%	40%	100%

*Note:* Bartlett's test *p* value 0.0003.

### 2.3 Statistical Analysis

We performed statistical analyses to investigate whether there was a correlation between the overall EQAP performance and specific technical details supplied by EQAP participants. We carefully reviewed the information regarding the workflows and protocols provided by these participants to ensure they contained conclusive data about the kits or tests they used. In cases where additional clarification was needed, we reached out to the participants to confirm or provide more specific information. We assessed the variables for normal distribution using the Brown‐Forsythe and Bartlett's test. All statistical analyses were carried out using GraphPad Prism 9.

## 3 Results

### 3.1 Testing Procedure

**FIGURE 2:**
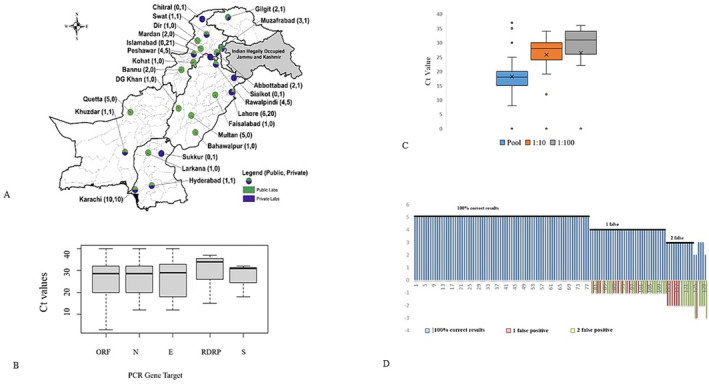
(A) Geographic map of SARS‐CoV‐2 EQA panel laboratories, (B) Number of correct, false positive, and false negative samples, (C) Comparison of Ct values with EQA panel samples concentration. (D) Comparison of Ct values with different commercial kits target genes. The highest Ct values are indicated by upper whisker, median values by middle quartile, and lowest Ct values by lower whisker.

A total of 141 laboratories from all over the country participated in EQA panels and reported results. The evaluation was based on four panels dispatched between August 2020 to December 2021, a geographical map of SARS CoV‐2 laboratories in country, including the Federal Capital, Punjab, Sindh, Balochistan, Khyber Pakhtunkhwa, AJK, and Gilgit Baltistan (Figure 2A).

Seventy‐eight (56%) of 141 enrolled laboratories reported accurate results for all five samples, thirty‐four (24%) reported four samples corrected and one false negative while 29 (20%) reported three or less correctly out of five samples (Brown‐Forsythe test; *p* = 0.0029) (Figure 2B).

Notably, only 21 result submissions 141 were based on the outcome of two or more test methods. The 120 remaining participants scored their results based on the outcome of a single method.

The risk of false‐negative tests increased significantly with lower SARS‐CoV‐2 concentrations.

(< 0.004, Brown‐Forsythe correlation test) (Figure 2C). Ct values of correctly tested SARS‐CoV‐2 samples increased with decreasing concentration.

Furthermore, false‐negative and false‐positive test results increased significantly with a change of SARS‐CoV‐2 commercial kit gene target (Brown‐Forsythe; *p* < 0.0004). However, the best‐performing test for SARS‐CoV‐2 S gene target produced noticeably better results (Figure 2D).

Eighty‐seven public sector laboratories (62%) out of 141 enrolled laboratories reported SARS‐CoV‐2 results. Out of which during all EQAP 1–4, 53 laboratories (53/87: 61%) reported 100% accurate results for all samples. In EQAP‐1, a total of 7 laboratories was enrolled out of which four (4/7: 57%) reported 100% results followed by two (2/7: 28%) showed 80% and only one laboratory had < 80%. During EQAP‐2, 24 laboratories (24/40: 60%) showed 100% of the results followed by five laboratories (5/40: 12%), whereas seven laboratories (7/40: 17%) exhibited < 80%. During EQAP‐3, three laboratories (3/7: 42%) reported 100% results, two (2/7: 28%) 80% results and two were < 80%. A total of 34 laboratories was enrolled in EQAP‐4 out of which 22 (22/34: 64%) and 15 (15/34: 44%) showed 100% and 80%, respectively, whereas only one laboratory reported < 80% result (Table 2).

**TABLE 2:** EQAP public laboratories scoring.

Regions	No. of lab‐reported results	No. (%) labs with 100%	No. (%) labs with 80%	No. (%) labs with < 80%
EQAP‐1	7	4	2	1
EQAP‐2				
Federal Capital	—	—	—	—
Punjab	13	7 (66)	1 (8.3)	3 (25)
Sindh	12	10 (83)	1 (8.3)	—
Balochistan	3	1 (33)	1 (33)	1 (33)
KPK	11	4 (36)	4 (36)	2 (18)
Gilgit Baltistan	1	1 (100)	—	—
AJK	3	1 (33)	1 (33)	1 (33)
Total	40	24	5	7
EQAP‐3				
Federal Capital	—	—	—	—
Punjab	2	1 (50)	1 (50)	
Sindh	—	—	—	—
Balochistan	1	—	—	1 (100)
KPK	2	1 (50)	—	1 (50)
Gilgit Baltistan	1	1 (100)	—	—
AJK	1		1 (100)	
Total	7	31	2	2
EQAP‐4				
Federal Capital	10	2 (20)	6 (60)	2 (20)
Punjab	18	13 (72)	3 (16)	2 (11)
Sindh	3	3 (100)	—	—
Balochistan	—	—	—	—
KPK	3	2 (66)	1 (33)	—
Gilgit Baltistan	—	—	—	—
AJK	—	—	—	—
Total	34	22	15	1

Fifty‐four (38%) out of 141 private sector enrolled laboratories reported SARS‐CoV‐2 results. Among EQA panels 2, 3, and 4, 16 out of 54 labs (30%) of the labs were reporting 100% results. Private sector laboratories were not enrolled in EQAP‐1 testing phase. In EQAP‐2, all eight enrolled laboratories reported accurate results for all samples. In EQAP‐3, a total of 23 laboratories (23/38: 61%) showed 100% of the results, while 14 (14/38: 37%) laboratories reported 80% results and only one lab reported > 80%. In EQAP‐4, out of total eight enrolled, seven laboratories (7/8: 87%) showed 100% of the results and the remaining one (1/8: 13%) represented 80% result (Table 3).

**TABLE 3:** EQAP private laboratories scoring.

Regions	No. of lab‐reported results	No. (%) labs with 100%	No. (%) labs with 80%	No. (%) labs with < 80%
EQAP‐1	—	—	—	—
EQAP‐2				
Federal Capital	3	3 (100)	—	—
Punjab	2	2 (100)	—	—
Sindh	2	2 (100)	—	—
Balochistan	1	1 (100)	—	—
KPK	—	—	—	—
Gilgit Baltistan	—	—	—	—
AJK	—	—	—	—
Total	8	8	—	—
EQAP‐3				
Federal Capital	8	5 (62.5)	3 (28)	—
Punjab	11	5 (60)	5 (35)	1 (7)
Sindh	7	4 (57)	3 (21)	—
Balochistan	1	1 (100)	—	—
KPK	3	1 (33)	2 (14)	—
Gilgit Baltistan	1	1 (100)	—	—
AJK	—	—	—	—
Total	31	23	14	1
EQAP‐4				
Federal Capital	—	—	—	—
Punjab	2	2 (100)	—	—
Sindh	2	2 (100)	—	—
Balochistan	1	1 (100)	—	—
KPK	2	1 (50)	1 (50)	—
Gilgit Baltistan	—	—	—	—
AJK	1	1 (100)	—	
Total	8	7	1	—

As performance in an EQA is the result of multiple stages in the diagnostic workflow, these stages consists of various factors such as extraction method and PCR kit used for testing, the type of commercial PCR assay used (gene target), its sensitivity, and more. In total, the EQA participants used 12 different extraction kits. Among the enrolled laboratories, the EQA performance was not significantly correlated to the type of extraction kit used Brown‐Forsythe (*p* = 0.4317) (data not shown).

An extensive variety of commercial PCR kits is used among the EQAP participating laboratories. According to the Brown‐Forsythe test (*p* = 0.4317), there was no significant correlation between the performance and any particular type of PCR assay (Table 1).

## 4 Discussion

The performance across this EQA exhibited variability. The majority of false results was observed in low‐concentration SARS‐CoV‐2 samples. This is noteworthy because infectious SARS‐CoV‐2 patients typically have high viral titers, especially during the initial stages of illness [8, 9, 10]. As a result, diagnosis, isolation and therapy for symptomatic patients, and contact tracing, consequently, may be delayed. In this context, having an optimized diagnostic sensitivity becomes crucial to identify patients who fall outside the ideal window for detection or in cases where sampling or transportation conditions are suboptimal [9, 11]. These findings agree with Kim et al., which showed that high sensitivity of direct antigenic tests can be achieved only for SARS‐CoV‐2 positive samples with a Ct value of ≤ 25 [10, 15].

Conducting diagnostic tests for emerging diseases such as SARS‐CoV‐2 in governmental and public labs significantly lengthens the TAT for logistical and analytical procedures [13]. EQAP in laboratory medicine is critical for evaluating the efficacy and status of diagnostic assays in diagnostic settings [2]. Diagnostic assay reproducibility and reliability are particularly important in clinical management and for public health purposes [5]. The Eastern Mediterranean Region of the WHO has faced significant challenges in responding to emerging infectious diseases, such as the COVID‐19 pandemic, these include weak health systems, limited surveillance and reporting, high population density, and mobility [1]. Low concentrations of the virus in clinical samples are frequently observed during later stages of infection, coinciding with seroconversion and a decrease in infectivity [16].

In the current study, EQA's overall performance was variable. All studies in COVID‐19 patients show a gradual decline in viral load over time, with positive detection occurring after the onset of symptoms [8]. Small viral loads in clinical samples are also common at later stages of infection, which coincide with seroconversion and a decrease in infectivity [8]. When a diagnosis is required for proper patient supervision and infection prevention measures, such as in hospitalized patients with a strong suspicion of SARS‐CoV‐2 infection but repeated rtRT‐PCR false negative test results, serological tests such as enzyme‐linked immunosorbent assays in diagnostic algorithms may decrease the likelihood of false‐negative tests [2].

The results of two or more test methods were used to score the remaining participants' results. Lower SARS‐CoV‐2 concentrations increased the risk of false‐negative tests significantly. Measured Ct values of accurately tested SARS‐CoV‐2 specimens increased as concentration decreased. A wide range of rtRT‐PCR methods was used among the participants in this EQA. The EQA performance was unrelated to the extraction kit type. In general, there was no significant correlation between performance and PCR assay type. Because transcription in coronaviruses varies across genomic and subgenomic regions, the target sites of the used assays may influence diagnostic sensitivity [8, 9]. However, the fraction of correct results did not vary significantly across genomic targets.

Laboratories' performance was based on their RNA extraction and amplification systems. Notably, the labs that reported false‐positive results used both extraction kits and rtRT‐PCR tests that other labs also utilized. Contamination issue rather than issues related to probe or primer was responsible for false positive results, so, false‐positive test results could be caused by contamination during sample handling and extraction or by lot‐specific contamination of rtRT‐PCR kits or oligonucleotides [17]. Extremes in temperature, humidity, and pH, as well as uncontrolled buffering capacity or ionic strength, can also lead to false positive results [18]. These conditions are not advised by the manufacturer. In any case, laboratories which reported false positive results must modify workflows to maintain good sensitivity, which was generally high among participants [13].

In Pakistan, this EQAP study is the first of its kind. Accordingly, its performance in a recently published SARS‐CoV‐2 EQA panel, which compares it to other first‐line, routine clinical laboratories, indicates that participant sensitivity in our EQAP was lower for some samples [2]. Given that sub‐optimal sensitivity in EQAs for molecular diagnostics of newly emerged viruses is not uncommon, both routine clinical laboratories and expert laboratories of public and private sectors performed well [3, 10, 19]. Noticeably, the results show that harmonized workflows, rather than specific extraction or rtRT‐PCR kits, can improve performance. Neither of the independent workflow components evaluated for this survey could significantly affect a laboratory's overall performance.

Follow‐up EQAs will be required to help national laboratories systematically improve and maintain diagnostic capabilities, while the need for a robust global detection capability necessitates global EQA programs. It is therefore advised to increase monitoring by utilizing all available public and private resources to fully achieve representativeness and increase sensitivity and quality reporting. This study highlights the risk of cross‐contamination in rtRT‐PCR. Vigilance will aid in avoiding critical laboratory response delays now and in future outbreak events.

## 5 Conclusion

EQA results with a range of assays are a suitable way to assess the performance of laboratories. These laboratories were the initial participants in the national SARS‐CoV‐2 surveillance program. The standardization of molecular laboratory detection methods will continue to be crucial in monitoring SARS‐CoV‐2 vaccine production and the requirement to provide reliable data to fill knowledge gaps in the epidemiology of SARS‐CoV‐2. Implementing quality assessment techniques for SARS‐CoV‐2 detection and genetic variability should play equally essential roles as the WHO SARS‐CoV‐2 surveillance continues to grow. Working procedures and quality control rules should be thoroughly followed in clinical practice. Our findings indicate that in resource‐constrained settings, EQA studies and on‐site assessments accompanied by guidance can help to improve such outcomes. In such cases, we believe that this model could be a valuable tool for laboratory strengthening.

## Author Contributions

**Nazish Badar:** conceptualization, formal analysis, funding acquisition, investigation, methodology, project administration. **Aamer Ikram:** funding acquisition, project administration, resources. **Muhammad Salman:** funding acquisition, project administration. **Sidra Saeed:** data curation, formal analysis. **Hamza Ahmed Mirza:** formal analysis, investigation, methodology. **Abdul Ahad:** data curation, formal analysis. **Asiya Ashraf:** methodology, writing–review and editing. **Umer Farooq:** formal analysis, investigation.

## Ethics Statement

The Internal Review Board of Pakistan NIH approved the severe acute respiratory infections (SARI) data collection and surveillance. Each participant provided formal written or verbal consent. Moreover, patient identities were kept confidential. The institutional board authorized the study upon review of the study protocols. The data form had a checkbox for recording the consent‐gathering process.

## Conflicts of Interest

The authors declare no conflicts of interest.

### Peer Review

The peer review history for this article is available at https://www.webofscience.com/api/gateway/wos/peer‐review/10.1111/irv.13316.

## Supporting information


**Figure S1.** Instructions and information EQA SARS CoV‐2.

**Table S1.** Sample of EQA panels with Ct values.

## Data Availability

All relevant data are within the paper tables and figure files.
