# Supplementary material for: External Quality Assessment Program for SARS‐COV‐2 Molecular Detection in Pakistan
Source: Influenza Other Respir Viruses. 2024 Jul 11;18(7):e13316. doi: 10.1111/irv.13316 (PMC11239755; doi:10.1111/irv.13316)
Supplement: Supplementary file 1 — Figure S1. Instructions and information EQA SARS CoV‐2. [file IRV-18-e13316-s002.pdf]

External Quality Assurance Panel for SARS-CoV-2

Instructions and Information_EQA-SARSCOV2-2020A

Package Composition:

05 EQA samples in 2ml cryovials containing 800ul each in triple packaging
Instructions for User

Upon receipt of the panel:

Inspect the contents of the EQA panel for completeness (05 samples in total) and for damage/broken or leaking samples. Do not use damaged, broken or leaking material for testing.

Please confirm receipt of the panel within 48 hours and notify the National Institute of Health, Islamabad through email.

If the panel is incomplete or samples are damaged by sending an e-mail to:

nazishbadar@yahoo.com

Safety warning:

SARS-CoV-2 EQA panel samples may contain infectious coronaviruses. Unpacking and all subsequent procedures involving open tubes should be performed inside a functional Class II biosafety cabinet (BSC-II)

Processing of the samples:

1. Each EQA sample is 800ul in volume and is clearly identified as EQA-SARSCOV2-2020- and sample number V1-V5.
2. Please process all samples following the RNA extraction and PCR protocols established by your laboratory.

Interpretation & Reporting:

1. Reporting information should be done electronically on the excel template**;** EQA-SARSCOV2-2020A-REPORT.xlsx
2. Results should be reported **with Ct values** for target viral genes as well as internal and positive control.
3. The results should be reported using the following terms(given in drop down menu in excel reporting template) i.e. positive, negative,invalid; inconclusive etc.) against each sample
4. The participating labs should also provide the **run file** along with the completed reporting template

Reporting of EQA Panel results:

Your EQA results should be submitted electronically [completed excel template EQA-SARSCOV2-2020A-REPORT.xlsx template and run data] to nazishbadar@yahoo.com within 7 days after receipt of the panel.

Additional Mandatory Information:

The user is expected to provide with the following details on the template in addition to sample results:

Laboratory name
Date of test run
Real-time PCR Thermocycler: Manufacturer
Real-time PCR Thermocycler: Model
Last calibration (if applicable)
RT-PCR kit Manufacturer:
RT-PCR kit Product Code:
RT-PCR kit Lot. No.
RT-PCR kit expiry:
Nucleic acid (RNA) Extraction kit method (Automated or Manual):
Automated Nucleic acid (RNA) Extraction
Nucleic Acid (RNA) Extraction kit Manufacturer:
Nucleic Acid (RNA) Extraction kit Product Code:
Nucleic Acid (RNA) Extraction kit Lot. No:

Assessment and Scoring

Maximum score	100 %
Passing score	80%

Penalties

Late reporting	- 10 %
