# Supplementary material for: External Quality Assessment Program for SARS‐COV‐2 Molecular Detection in Pakistan
Source: Influenza Other Respir Viruses. 2024 Jul 11;18(7):e13316. doi: 10.1111/irv.13316 (PMC11239755; doi:10.1111/irv.13316)
Supplement: Supplementary file 2 — Table S1. Sample of EQA panels with Ct values. [file IRV-18-e13316-s001.docx]

**Supplementary material**

**Table 1S. Sample of EQA Panels with Ct Values.**

| **Sample No.** | **Ct value** |
| --- | --- |
| 1(Pool) | 18 |
| 2(1:10) | 30 |
| 3(1:100) | 35 |
| 4 (Buffer/Negative) | 0 |
| 5(Negative) | 0 |
